# Diagnosis and Treatment Strategies of Eosinophilic Esophagitis: A Nationwide Survey

**DOI:** 10.3390/diagnostics16213505

**Published:** 2026-10-26

**Authors:** Fahmi Shibli, Anat Nevo Shor, Amir Mari, Ori Segol, Noam Zevit, Jawad Hindy, Ram Dickman

**Affiliations:** 1 Institute of Gastroenterology and Liver Diseases, Emek Medical Center, Rappaport Faculty of Medicine, Technion-Israel Institute of Technology, Afula 1834111, Israel; 2 Department of Gastroenterology and Liver Diseases, Soroka Medical Center, Faculty of Medicine, Ben-Gurion University of the Negev, Beer Sheva 8410501, Israel; 3 Department of Gastroenterology, Nazareth EMMS Hospital, Faculty of Medicine in the Galilee, Bar-Ilan University, Nazareth 1610000, Israel; 4 Institute of Gastroenterology and Liver Diseases, Carmel Medical Center, Rappaport Faculty of Medicine, Technion-Israel Institute of Technology, Haifa 3436212, Israel; 5 Institute of Gastroenterology, Nutrition, and Liver Diseases, Schneider Children’s Medical Center, Gray Faculty of Medical and Health Sciences, Tel Aviv University, Petah Tikva 4920235, Israel; 6 Institute of Gastroenterology and Liver Diseases, Bnai Zion Medical Center, Rappaport Faculty of Medicine, Technion-Israel Institute of Technology, Haifa 3104802, Israel; 7 Division of Gastroenterology, Rabin Medical Center, Beilinson Campus, Gray Faculty of Medical and Health Sciences, Tel Aviv University, Petah Tikva 4941492, Israel

**Keywords:** eosinophilic esophagitis, diagnostic algorithm, elimination diet, endoscopic surveillance, adherence

## Abstract

**Background/Objectives:** Eosinophilic esophagitis (EoE) is an increasingly recognized chronic immune-mediated inflammatory disease of the esophagus. Data on the real-world diagnosis and management practices among gastroenterologists in Israel is scarce. This study aimed to describe current diagnostic and treatment practice patterns for eosinophilic esophagitis among Israeli gastroenterologists, and to identify areas of concordance with, and variability relative to, international recommendations. **Methods:** A nationwide, cross-sectional, internet-based survey was conducted among gastroenterologists from the Israeli Gastroenterology Association (IGA) and the Israeli Society of Pediatric Gastroenterology, Hepatology and Nutrition between February and April 2024. We used a structured 28-item questionnaire covering physician demographics, diagnostic practices and treatment strategies of EoE. Responses were analyzed for the entire cohort and compared across subgroups based on physician characteristics. **Results:** Of the 540 eligible gastroenterologists, 114 (22%) completed the survey. Almost all responders (97%) would obtain esophageal biopsies in the case of dysphagia even without typical endoscopic findings. Similarly, 94% of the responders would obtain esophageal biopsies in the case of typical endoscopic findings even without dysphagia. About 86 (75%) were familiar with the Endoscopic Reference Score (EREFS) system. Proton pump inhibitors were the most prescribed first-line treatment (92%), followed by dietary interventions (40%) and topical steroids (17%). As compared with adult gastroenterologists, pediatric gastroenterologists were more likely to obtain biopsies, use the EREFS and initiate treatment before histologic confirmation was obtained. Overall, practices showed broad concordance with international recommendations (>75% of respondents). **Conclusions:** In this cross-sectional survey, it was demonstrated that diagnostic and treatment practices for EoE among Israeli gastroenterologists were broadly concordant with international recommendations. However, several areas of inconsistency across different provider subgroups were identified. These gaps can be managed through educational programs that will improve standardization of management in EoE.

## 1. Introduction

Eosinophilic esophagitis (EoE) is a chronic, immune-mediated inflammatory disease of the esophagus. EoE affects both adults and children; however, presentation is different due to the progression in esophageal remodeling. In children, EoE is characterized by vomiting, dysphagia, and feeding difficulties. In adults, long-standing EoE may be presented with dysphagia, food impaction and esophageal strictures that require dilations. Diagnosis of EoE requires definite histologic evidence demonstrating at least 15 eosinophils per high-power field, typical symptoms of esophageal dysfunction and the absence of other conditions associated with esophageal eosinophilia [1]. EoE is a relatively new disease with rising incidence, attributed to increased clinical awareness along with a true increase in incidence [2,3].

The endoscopic appearance of the esophagus is not specific, as in 25% of cases in which esophageal biopsies reveal significant esophageal eosinophilia, endoscopic appearance is completely normal. Therefore, a high clinical index of suspicion and correct methodology of obtaining histological sampling during endoscopy are crucial [4].

EoE treatment has been markedly evolved in recent years. Current therapies include proton pump inhibitors (PPIs), topical steroid formulations, dietary modifications, endoscopic dilation and biological agents [5,6]. Due to the paucity of high-quality evidence from randomized controlled clinical trials (RCTs) comparing various treatments for EoE and the lack of a standardized system to assess response, there is a need for evidence-based clinical guidelines for treatment and surveillance strategies.

Thus, the first aim of this study was to describe the current diagnosis and management practices for EoE among Israeli gastroenterologists, and to identify specific areas in which these practices are concordant with, or diverge from, international recommendations. Throughout this manuscript, real-world diagnostic and treatment practices are reported descriptively; the degree of concordance between these reported practices and pre-defined, guideline-based recommendations (detailed in the Methods) is then highlighted to identify specific targets for future educational efforts. Based on the results of this survey, we intend to implement a future national educational program through courses and then to repeat the survey.

## 2. Materials and Methods

We conducted an online survey among all adult gastroenterologists and pediatric gastroenterologists registered as members of the Israeli Gastroenterology Association (IGA) and the Israeli Society of Pediatric Gastroenterology, Hepatology and Nutrition (ISPGHN). A 28-question, multiple-choice anonymous questionnaire was electronically delivered by email. The questionnaire was developed by the study investigators, all of whom are practicing adult or pediatric gastroenterologists with expertise in EoE, based on the clinical recommendations detailed below, and was reviewed internally by the author panel for clarity and content coverage before distribution. The survey was built and administered using Google Forms. The questionnaire was not pilot-tested on an external sample but underwent internal validation by the authors, all practicing adult or pediatric gastroenterologists, prior to distribution. The survey link was sent once to each recipient via the existing institutional mailing lists of the IGA and ISPGHN, rather than to individually identified physicians; as a single invitation was sent per list and responses were anonymous, duplicate submissions from the same recipient could not be formally prevented by the platform, though we have no indication that this occurred. The exact number and timing of reminder emails, if any, were not systematically recorded and cannot be reported with confidence. All 28 items were mandatory, and respondents could not skip a question; consequently, no partially completed questionnaires were submitted, and no questionnaires were excluded from analysis for incompleteness. The full questionnaire (Hebrew, with an English translation) is provided as Supplementary File S1. Questions explored three areas of interest: physician demographics, diagnostic practices for EoE, and treatment strategies for EoE. The questionnaire consisted of 27 multiple-choice questions and one optional, open-ended, free-text question in Hebrew inviting additional comments; responses to this open-ended item were not subjected to systematic qualitative or quantitative analysis and are not reported here.

The questionnaire content was developed based on recommendations from internationally recognized guidelines and consensus statements on EoE available at the time of its design, representing both North American and European perspectives: the American Gastroenterological Association/Joint Task Force on Allergy-Immunology Practice Parameters clinical guideline [5], the American Society for Gastrointestinal Endoscopy Consensus Conference on the endoscopic approach to EoE [4], the European Society of Gastrointestinal Endoscopy tissue-sampling guideline [7], and the literature on proton pump inhibitor-responsive esophageal eosinophilia [8]. Since the time of the survey, the diagnostic and management framework for EoE has been further updated by the International Consensus Diagnostic Criteria from the AGREE conference [9] and, for a joint adult-and-pediatric European perspective, by the British Society of Gastroenterology/British Society of Paediatric Gastroenterology, Hepatology and Nutrition consensus guidelines [10]; the practice patterns reported below are discussed in relation to both the original guideline framework used to design the questionnaire and these subsequently updated recommendations. Reported practices were then compared descriptively to the corresponding recommendation from these sources for each of the following clinical scenarios: obtaining esophageal biopsies regardless of the endoscopic appearance in a symptomatic patient; the number and location of biopsies taken; familiarity with and use of the EREFS classification system; an 8-week trial of proton pump inhibitor therapy as first-line management; discontinuation of proton pump inhibitor therapy before biopsy in suspected EoE; and the criteria used to define treatment response. For each of these items, we report the proportion of respondents whose reported practice was concordant with the corresponding recommendation.

Gastroenterologists included in this study were classified as either being a senior (board certified in gastroenterology) or a gastroenterology trainee. We collected data on: practicing site (academic medical center or a community medical center), experience (number of practicing years as a gastroenterologist), age of patients (below or over 18 years) and the burden of EoE (number of visits on a regular basis every month).

The survey was distributed to all adult and pediatric gastroenterologists registered as members of the IGA and ISPGHN at the time of the study (540 individuals); of these, 114 (22%) completed the questionnaire. The survey invitation was distributed as a single mass email through the existing institutional mailing lists of the IGA and ISPGHN rather than to individually tracked physician addresses; consequently, per-recipient delivery confirmation, bounce rates, and the number or timing of reminder emails were not systematically recorded and cannot be reported with confidence, and a conventional individual-level invitation/response flow diagram could not be constructed. Individual-level demographic data for nonresponding members (e.g., age, sex, region, specialty, or practice setting) were not available to us, so a formal comparison between respondents and the full eligible population could not be performed; this limitation, and its implications for potential nonresponse bias, is discussed further in the Strengths and Limitations section.

## 3. Statistical Analyses

The data was analyzed for the total sample, and the demographic analysis compared subgroups by eight different criteria [gender, age groups (30–39 years, 40–49 years, >50 years), geographic region (north, center, south/Jerusalem), specialty (adult GI vs. pediatric GI), years in practice (up to 5 years, 6–10 years, >10 years), practice settings (hospital based, community based, both), subspeciality (neurogastroenterology and motility, advanced endoscopy, neither), and gastroscopy load (low, medium, high)]. The maximum sampling error was ± 8% at a 95% confidence level. Crosstabs significance was calculated using the Pearson chi-square test for comparisons in which all expected cell counts were five or greater; for comparisons involving smaller subgroups (e.g., community-based practice, *n* = 10; pediatric specialists, *n* = 22; and other subgroups with small cell counts), Fisher’s exact test was used instead, since the chi-square approximation is unreliable under these conditions. In multiple comparisons (demographic analysis), Bonferroni correction was applied: for each of the eight demographic criteria examined, pairwise subgroup comparisons were corrected for multiple testing using a Bonferroni-adjusted significance threshold; for criteria with three subgroups (yielding three pairwise comparisons), this corresponded to an adjusted alpha of approximately 0.017 (0.05/3). Odds ratios and 95% confidence intervals are reported alongside *p*-values for key comparisons to convey effect size, as detailed in Section 4.

## 4. Results

### 4.1. Respondent Characteristics

Of the 114 respondents, the majority practiced adult gastroenterology (81%), with the remainder practicing pediatric gastroenterology (19%). Eighty-seven percent were senior, board-certified gastroenterologists and 13% were gastroenterology trainees. Sixty-three percent of respondents were male and 37% female. Years of clinical experience were fairly evenly distributed: 39% had been in practice for five years or less, 25% for 6–10 years, and 36% for more than a decade. Practice setting was hospital-based in 43% of respondents, community-based in 9%, and a combination of both in 48%. By subspecialty, 31% identified as neurogastroenterology/motility and 18% as advanced endoscopy, while the remaining 53% reported neither. Respondents practiced across all regions of the country, most commonly in the North (40%) and Center (39%), with a smaller proportion in the South/Jerusalem area (25%). EoE-related gastroscopy workload was low in 19% of respondents, medium in 44%, and high in 37%. A full breakdown of respondent characteristics is presented in Table 1.

### 4.2. EoE Diagnosis

**Figure 1:**
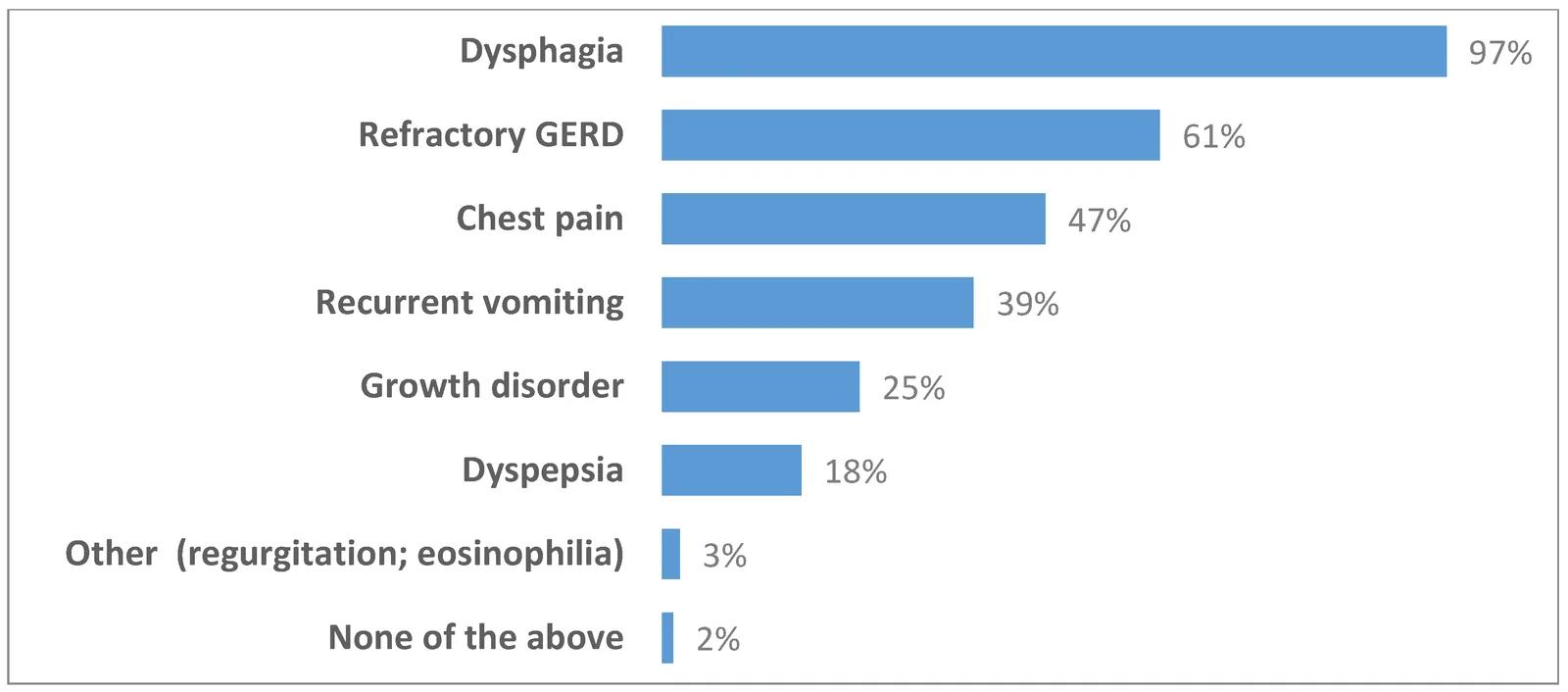
Indications for esophageal biopsy in patients with a normal endoscopic appearance (% of physicians).

Almost all responders (97%) stated that they would obtain esophageal biopsies in the case of dysphagia even without typical endoscopic findings. Esophageal biopsies were also recommended in patients with a normal appearing esophagus in the setting of refractory GERD (61%), chest pain (47%), recurrent vomiting (39%), growth disorder (25%), and dyspepsia (18%) (Figure 1).

**Figure 2:**
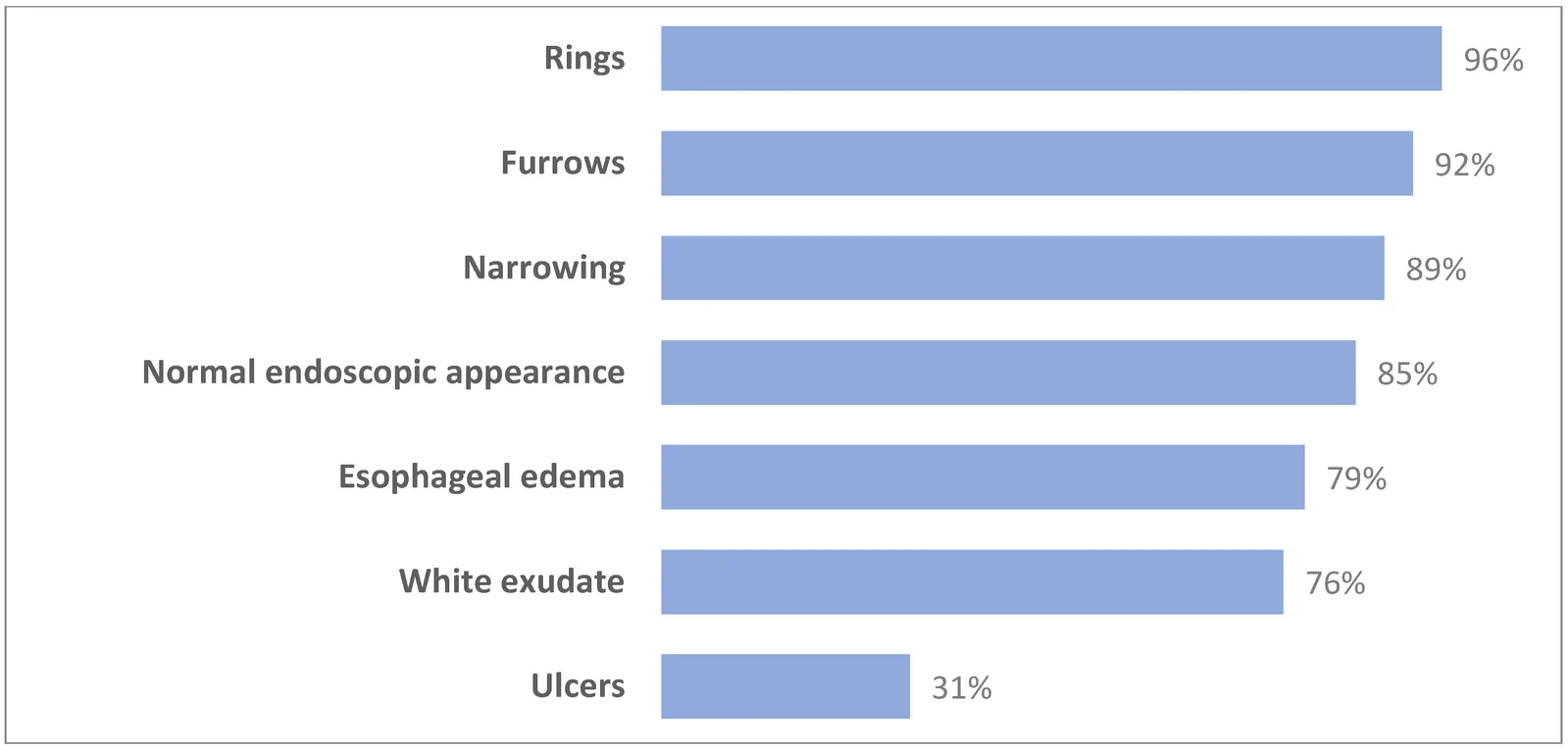
Endoscopic findings expected in eosinophilic esophagitis (% of physicians).

In the setting of patients with endoscopic findings suspicious for EoE but without dysphagia, 94% of responders stated they would obtain esophageal biopsies as well. The most common endoscopic findings for suspected EoE were rings (96%), followed by furrows (92%), narrowing (89%), normal endoscopic appearance (85%), edema (79%), and/or white exudate (76%) (Figure 2). Ulcers were mentioned only by 31% of the responders. About 75% of responders were familiar with the EREFS system for evaluating the severity of endoscopic findings in EoE.

**Figure 3:**
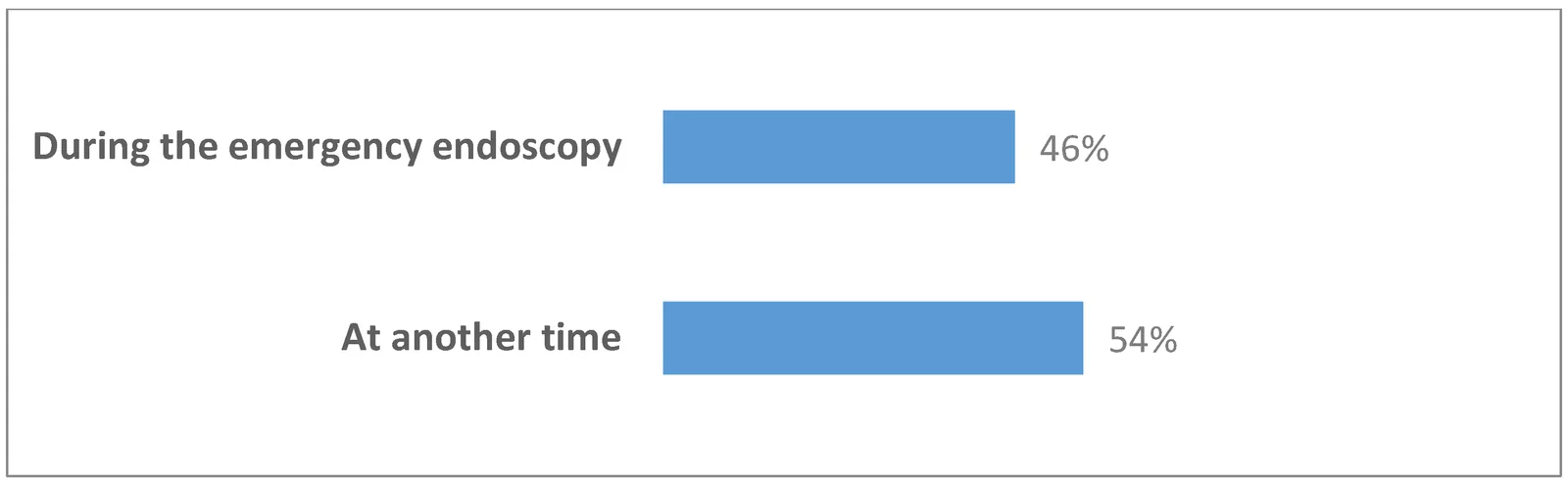
Timing of esophageal biopsy in acute food impaction (% of physicians).

In the case of acute food impaction, 46% of responders were in favor of taking esophageal biopsies, while 54% were against this approach (Figure 3).

While most respondents (65%) would take at least six biopsies, 29% would take four biopsies, and 6% would take only two biopsies. Biopsies for EoE were primarily taken from the middle esophagus (91% of physicians) and lower esophagus (86%), with 64% also taking biopsies from the upper esophagus and 20% from the stomach. When taking biopsies from multiple sites, 86% of specialists would send the samples in separate biopsy containers.

Less than half of the respondents (47%) recommended stopping PPI treatment before taking esophageal biopsies for suspected EoE.

### 4.3. EoE Treatment

**Figure 4:**
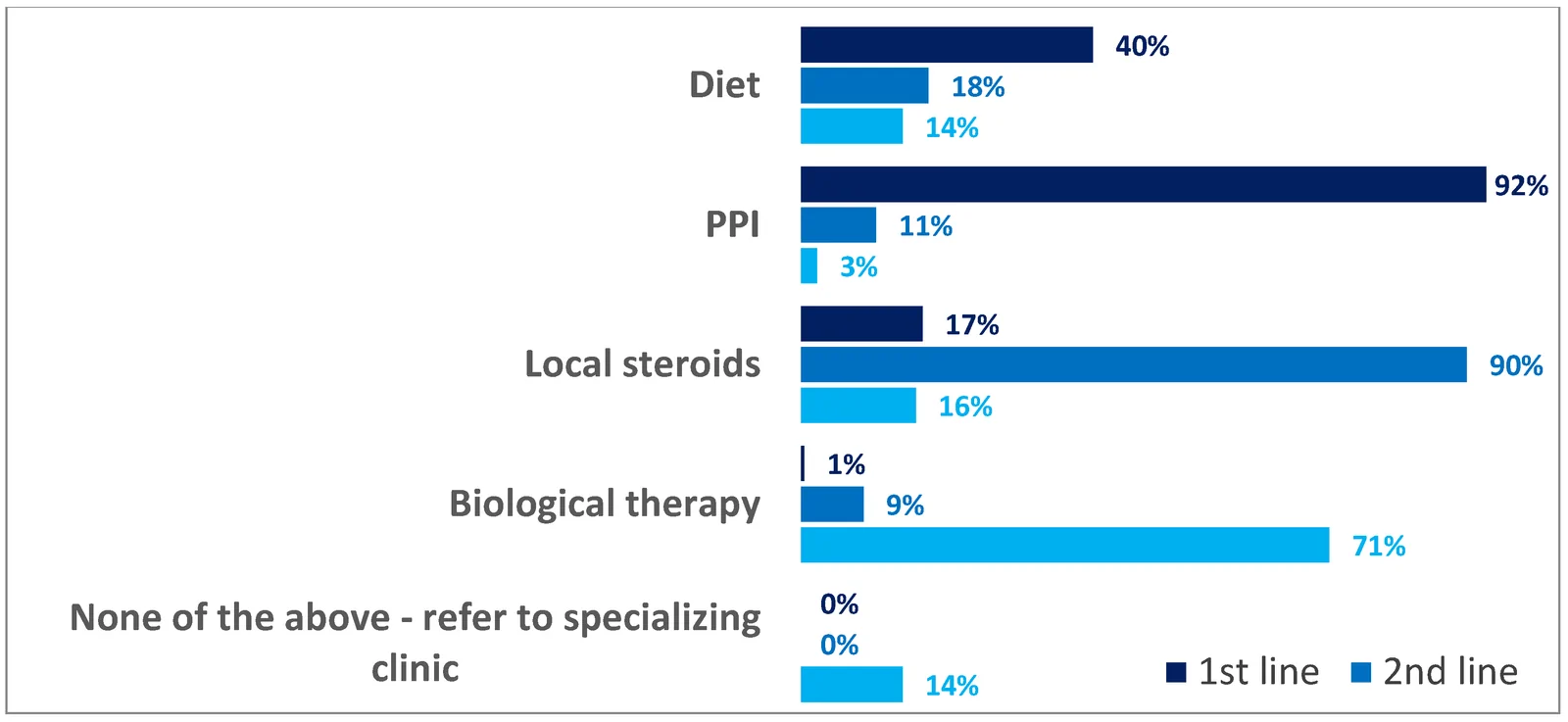
Preferred treatment strategy by line of therapy (% of physicians).

In a patient with an endoscopic appearance suspicious for EoE, 47% of GI specialists would initiate treatment before receiving the histological results. As first-line treatment for EoE, 92% would prescribe PPI, 40% would also prescribe a food elimination diet (FED) (1FED 20%, 2FED 36%, 4FED 8%, and 6FED 36%), and 17% would give topical steroids (Figure 4). In case of first-line treatment failure, 90% would prescribe topical steroids, 18% FED therapy, 11% PPI, and 9% biological therapy. In cases of treatment failure after two lines of therapy, 71% would prescribe biological therapy, 16% topical steroids, 14% FED, and 14% would not treat further but refer the patients to an EoE specialty clinic.

For each treatment line, respondents were permitted to select more than one option, since combination therapy (e.g., concurrent PPI and dietary elimination) is commonly used in clinical practice; consequently, the percentages reported for each line of treatment do not sum to 100%.

When selecting dietary treatment for EoE, 56% of GI specialists would choose a one or two food elimination diet, 36% would start with a six-food elimination diet, and 8% with a four-food elimination diet. Treatment response was defined by 90% of the specialists as a combination of clinical and histological improvement.

Forty percent of GI specialists referred every new EoE patient to an allergologist, while the remainder referred only if the patient had an allergic background such as allergies, atopic dermatitis, or asthma. The majority of GI specialists (75%) would repeat a gastroscopy after three months of treatment to assess endoscopic appearance and histological response. For an asymptomatic EoE patient, 63% of specialists would recommend annual endoscopic follow-up, and 24% would recommend follow-up every 6 months or less.

### 4.4. Demographic Differences

Pediatric GI specialists exhibited a different approach in several aspects compared to adult GI specialists. All pediatric GI specialists would take a biopsy when the endoscopic appearance was suspicious for EoE, even without patient complaints (100% vs. 92%). More pediatric GI specialists were familiar with EREFS (96% vs. 71%), would take six or more biopsies for EoE (82% vs. 61%), send biopsies in separate containers (100% vs. 83%), stop PPI treatment before a gastroscopy for suspected EoE, (73% vs. 41%), and start treatment before receiving histological results (68% vs. 42%). However, almost a third of pediatric GI specialists would refer EoE patients to allergologist (32% vs. 41%).

Familiarity with EREFS was higher among GI specialists in the Center region of Israel compared to the North (89% vs. 59%, *p* < 0.01). Community-based physicians showed lower familiarity with EREFS compared to hospital-based or both (20% vs. 78% and 84%, *p* < 0.01), and were less likely to stop PPI before gastroscopy for suspected EoE (10% vs. 47% and 55%, *p* < 0.05). Neurogastroenterologists demonstrated higher EREFS compared to general GI and other subspecialities (97% vs. 60–69%, *p* < 0.01). Other subspecialities prescribed PPI as first-line therapy more often than advanced endoscopy specialists (98% vs. 80%, *p* < 0.05), while the difference compared with neurogastroenterologists (98% vs. 89%) did not reach statistical significance (*p* = 0.06). Younger doctors (age 30–39 years) were less likely to refer EoE patients to allergologist compared to more experienced colleagues (27% vs. 40–48%), but more frequently used biological therapy as a third-line treatment compared with colleagues aged 40–49 years (89% vs. 65%, *p* < 0.05); the difference compared with colleagues aged 50 years and older (89% vs. 68%) did not reach statistical significance (*p* = 0.08)**.** Given the small size of some subgroups (e.g., community-based physicians, *n* = 10), all pairwise comparisons above were assessed using Fisher’s exact test, and effect sizes are reported as odds ratios (ORs) with 95% confidence intervals (CIs): EREFS familiarity, Center vs. North region, OR 5.49 (95% CI 1.83–16.50); EREFS familiarity, community vs. hospital-based, OR 0.07 (0.01–0.39), and community vs. both, OR 0.05 (0.01–0.27); stopping PPI before gastroscopy, community vs. hospital-based, OR 0.13 (0.01–1.07), and community vs. both, OR 0.09 (0.01–0.78); EREFS familiarity, neurogastroenterology vs. neither subspecialty, OR 15.38 (1.96–120.81), and vs. advanced endoscopy, OR 22.67 (2.56–200.63); PPI as first-line therapy, general GI vs. advanced endoscopy, OR 15.00 (1.57–143.70); biologic use as third-line therapy, age 30–39 vs. 40–49 years, OR 4.20 (1.10–16.07). The wide confidence intervals for several of these estimates reflect the small subgroup sizes and should be interpreted with caution.

## 5. Discussion

This nationwide survey, of both adult and pediatric gastroenterologists in Israel, provides valuable insights into the current diagnosis and treatment strategies for eosinophilic esophagitis (EoE). The study highlights areas of consensus as well as variations in practice patterns between adult and pediatric gastroenterologists, and across different regions and subspecialties (neurogastroenterology, advanced endoscopy, neither/general GI). These findings are broadly consistent with previously reported international survey data: for example, a survey of United States gastroenterologists similarly found that management of EoE often diverges from published recommendations and is not uniformly patient-centered, with marked differences in practice between academic and community settings [11]. Taken together, these observations suggest that discordance between guideline-based recommendations and real-world practice is not unique to Israel, but reflects a broader, international gap between consensus guidance and everyday clinical practice that likely benefits from continued education and implementation efforts.

One of the key findings of this survey is the high level of awareness among Israeli gastroenterologists regarding the importance of obtaining biopsies for the diagnosis of EoE in a symptomatic patient even in the absence of typical EoE endoscopic findings. Another key finding is the routine of obtaining biopsies for the diagnosis of EoE in an asymptomatic patient in the presence of typical or suspicious endoscopic findings for EoE. These practices align with current international guidelines, which emphasize the need for histological confirmation of EoE, given the variable and nonspecific clinical presentation and endoscopic features of this condition [4,7]. However, there appears to be some variability in the number and location of biopsies taken for the diagnosis of EoE. While most respondents (65%) indicated that they would take at least six biopsies for suspected EoE, a significant proportion (29%) would take only four biopsies, and a small minority (6%) would take only two biopsies. This variability in biopsy practices may reflect the lack of clear consensus in the literature regarding the optimal number and location of biopsies required for the diagnosis of EoE [12]. The survey revealed that the majority of gastroenterologists in Israel are familiar with the endoscopic findings associated with EoE, such as rings, furrows, narrowing, and edema.

Another key finding of this survey is the use of PPIs as first-line therapy for EoE (92%). These practices also align with current international guidelines which recommend a trial of PPI therapy for at least 8 weeks as the initial management strategy for EoE [8].

However, the survey also revealed that less than half of the respondents (47%) routinely recommend stopping PPI treatment before obtaining biopsies for suspected EoE. This practice may lead to delayed or missed identification of PPI-responsive cases. PPI-responsive esophageal eosinophilia (PPI-REE) was historically regarded as a clinical entity distinct from classic EoE; however, studies have shown that EoE and PPI-REE share similar clinical, endoscopic, and histological features [13]. Consequently, the updated international consensus diagnostic criteria no longer regard PPI-responsiveness as distinguishing a separate disease and now classify PPI-REE within the EoE spectrum, with a PPI trial retained as a therapeutic rather than a diagnostic step [8,9].

In terms of second- and third-line treatment options for EoE, the survey showed that local topical steroids and biological therapy are the most commonly preferred agents among Israeli gastroenterologists. This finding is consistent with the growing body of literature supporting the efficacy and safety of topical corticosteroids and targeted biologic therapies for the management of EoE [14,15]. However, the survey also highlighted some variations in practice patterns regarding the use of dietary therapy for EoE. While more than half of the respondents (56%) indicated that they would choose a one- or two-food elimination diet as their preferred dietary approach, a significant proportion (36%) would start with a more restrictive six-food elimination diet. This variability may reflect the lack of standardization and limited evidence base for dietary therapy in EoE, as well as practical challenges related to patient adherence and quality of life with dietary therapy [16,17].

The survey also provided some interesting insights into the referral patterns and follow-up practices for EoE. Only 40% of respondents indicated that they refer all new EoE patients to an allergologist, while the majority do not or refer only in the case of atopy. This finding suggests that there is room for improved collaboration and coordination of care between gastroenterologists and allergologists in the management of EoE. Additionally, the survey revealed some variability in the frequency and duration of endoscopic follow-up for EoE patients as most respondents recommending annual surveillance for asymptomatic patients on treatment.

A striking finding of this survey was the significant differences in practice patterns between adult and pediatric gastroenterologists. Pediatric gastroenterologists were more likely to obtain biopsies (particularly 6) for suspected EoE and to send them in separate containers. Pediatric gastroenterologists were more familiar with the EREFS scoring system and the recommendation to stop PPI therapy before obtaining biopsies for EoE. These differences may reflect a higher experience with EoE as incidence and prevalence of EoE in pediatric populations is much higher as compared to adults [3].

The finding that more pediatric gastroenterologists would start treatment before receiving histological results (68% vs. 42%) may be related to their greater emphasis that early diagnosis and aggressive management will prevent long-term complications such as esophageal remodeling and stricture formation [18,19].

The survey also highlighted some regional and subspecialty variations in EoE management practices, again, highlighting the need for continuing medical education on EoE in community-based gastroenterologist, especially in the periphery in Israel.

## 6. Strengths and Limitations of the Study

Major strengths of this survey include its broad geographic coverage, the inclusion of both adult and pediatric specialists and the systematic comparison of reported practices with international recommendations using appropriate statistical methodology. Limitations include a relatively low response rate (22% of the 540 eligible IGA/ISPGHN members) that may affect the accuracy of our findings, as physicians who did not respond may practice differently from those who did. We were not able to formally compare the demographic characteristics of respondents with those of the full eligible membership, as individual-level data on nonrespondents were not available to us; this limits our ability to quantify the direction and magnitude of potential nonresponse bias. However, because our respondent sample spanned a broad range of regions, practice settings, subspecialties, and experience levels broadly reflective of the national gastroenterology workforce, we believe major bias is unlikely, though it cannot be excluded, and the findings should be interpreted as describing practice patterns among engaged, survey-responsive gastroenterologists rather than the entire eligible population. As with any single-country survey, some of the specific practice patterns identified here may not be directly generalizable to gastroenterologists practicing under different healthcare systems or guideline frameworks; however, the guideline-based framework used for comparison is internationally applicable, and the educational gaps identified may be relevant to other settings facing similar variability in EoE management. In addition, as a cross-sectional survey, this study captures practice patterns at a single time point and does not include a pre-/post-educational intervention design; it therefore cannot establish whether a structured educational program would improve standardization of care. As noted above, we plan to use these findings to design a national educational program and to repeat this survey afterward, which will allow for a direct pre- and post-education comparison and a more definitive assessment of the impact of education on practice standardization. Despite these limitations, this study provides valuable baseline data on EoE practices in Israel and identifies specific areas where educational interventions may improve concordance with international recommendations.

## 7. Conclusions

This is the first nationwide survey that comprehensively describes EoE diagnostic and management practice patterns among Israeli gastroenterologists. Practices showed broad concordance with international recommendations in several key areas. However, the survey also identified specific areas of variability among providers, where continued educational interventions may help address unmet needs. Future research should focus on evaluating the impact of these practice variations on patient outcomes, as well as identifying strategies to improve the quality and consistency of EoE management practices in Israel and beyond.

## Figures and Tables

**Table 1:** Baseline characteristics of survey respondents (*N* = 114).

Characteristic	*n* (%)
Total respondents	114 (100)
**Specialty**	
Adult gastroenterology	92 (81)
Pediatric gastroenterology	22 (19)
**Professional Level**	
Senior/board-certified gastroenterologist	99 (87)
Gastroenterology trainee	15 (13)
**Gender**	
Male	72 (63)
Female	42 (37)
**Years in Practice**	
≤5 years	44 (39)
6–10 years	29 (25)
>10 years	41 (36)
**Practice Setting**	
Hospital-based	49 (43)
Community-based	10 (9)
Both	55 (48)
**Subspecialty**	
Neurogastroenterology/motility	35 (31)
Advanced endoscopy	20 (18)
Neither	61 (53)
**Geographic Region**	
North	46 (40)
Center	44 (39)
South/Jerusalem	28 (25)
**EoE-Related Gastroscopy Workload**	
Low	22 (19)
Medium	50 (44)
High	42 (37)

Percentages are calculated relative to the total sample (*N* = 114). Geographic region and subspecialty totals sum to slightly more than 114 in the source dataset, likely reflecting a small number of respondents classified under more than one category.

## Data Availability

The data presented in this study are available on request from the corresponding author.

## Supplementary Materials

The following supporting information can be downloaded at https://www.mdpi.com/article/10.3390/diagnostics16213505/s1. The original 28-item survey questionnaire (Hebrew, with an English translation) is available as Supplementary File S1.
