# Supplementary material for: Diagnosis and Treatment Strategies of Eosinophilic Esophagitis: A Nationwide Survey
Source: Diagnostics (Basel). 2026 Oct 26;16(21):3505. doi: 10.3390/diagnostics16213505 (PMC13761589; doi:10.3390/diagnostics16213505)
Supplement: Supplementary file 1 [file diagnostics-16-03505-s001.zip › diagnostics-4595845-supplementary.pdf]

Supplementary file S1

סקר דלקת אאוזינופילית של הוושט

Eosinophilic Esophagitis (EoE)

סקר אבחון, טיפול ומעקב בחולים עם דלקת אאוזינופילית של הוושט בקרב גסטרואנטרולוגים.

כל השאלות מוגדרות כחובה בטופס המקורי. יש לבחור תשובה אחת, אלא אם צוין אחרת.

1. מין:

☐ זכר

☐ נקיבה

☐ לא מעוניין.ת לענות

2. לאיזה קבוצת גיל את/ה משתייך/ת?

☐ 20-29

☐ 30-39

☐ 40-49

☐ 50-59

☐ מעל 60

3. אני גסטרואנטרולוג שמטפל ב:

☐ מבוגרים

☐ ילדים

4. באיזה שלב של הקריירה את/ה נמצא?

☐ מומחה/ית

☐ מתמחה

5. במידה ומומחה/ית: מה מספר שנות ותק כגסטרואנטרולוג/ית מומחה/ית?

☐ 0-5

☐ 6-10

☐ מעל 10

6. היכן את/ה עובד/ת- בבית חולים, בקהילה או בשניהם?

☐ בית חולים

☐ קהילה

☐ שניהם

7. באיזה אזור בארץ את/ה עובד/ת?

ניתן לסמן יותר מתשובה אחת.

☐ צפון

☐ דרום

☐ מרכז

☐ איזור ירושלים

8. תחום העניין במקצוע הגסטרואנטרולוגיה:

ניתן לסמן יותר מתשובה אחת.

☐ נוירוגסטרואנטרולוגיה

☐ מחלות מעי דלקתיות

☐ אנדוסקופיה מתקדמת

☐ תזונה

☐ כבד

☐ ממאירות

☐ כללי

9. מהו מספר הגסטרוסקופיות אותן את/ה מבצע/ת בחודש בממוצע?

☐ פחות מ 10

☐ 10-19

☐ 20-39

☐ 40 ויותר

10. באילו מהמצבים הבאים היית/ה בוחר/ת ליטול ביופסיה מהוושט כאשר המראה האנדוסקופי תקין לחלוטין?

ניתן לסמן יותר מתשובה אחת.

☐ דיספגיה

☐ כאבים בחזה

☐ צרבת רפרקטורית

☐ הקאות חוזרות

☐ דספפסיה

☐ הפרעה בגדילה

☐ אף אחד מהנ"ל

☐ אחר: ___________________________

11. האם היית/ה נוטל/ת ביופסיה מהוושט במטופל עם ממצאים המעלים חשד ל EoE אך ללא תלונות רלוונטיות?

☐ כן

☐ לא

12. אילו ממצאים היית/ה צופה באנדוסקופיה שיכולים להופיע ב EoE?

ניתן לסמן יותר מתשובה אחת.

☐ מראה אנדוסקופי תקין

☐ בצקת בושט

☐ תפליטים לבנים

☐ טבעות (Rings)

☐ תלמים (Furrows)

☐ היצריות

☐ כיבים

13. האם את/ה מכיר/ה את מערכת הניקוד EREFS להערכת אנדוסקופית של חומרת ה EoE?

☐ כן

☐ לא

14. האם במצב של חסימת מזון אקוטית (Food impaction) את/ה נוהג/ת ליטול ביופסיות מהוושט, ואם כן - מתי?

☐ בזמן האנדוסקופיה הדחופה

☐ במועד אחר

☐ כלל לא

15. כמה ביופסיות את/ה לוקח/ת מהוושט בחשד ל EoE?

☐ 2 ביופסיות

☐ 4 ביופסיות

☐ 6 ביופסיות ויותר

16. מאילו אזורים בוושט את/ה לוקח/ת ביופסיות במקרה של חשד ל EoE?

ניתן לסמן יותר מתשובה אחת.

☐ ושט תחתון

☐ ושט אמצעי

☐ ושט עליון

☐ קיבה

17. במקרה של חשד ל EoE, במידה ובחרת/ה ליטול ביופסיות מיותר מאתר אחד, כיצד תשלח/י את הדגימות?

☐ במיכל אחד

☐ במיכלים נפרדים

18. האם את/ה ממליץ/ה על הפסקת טיפול ב PPI לפני גסטרוסקופיה בחשד ל EoE?

☐ כן

☐ לא

19. במטופל עם מראה אנדוסקופי החשוד ל EoE , האם היית/ה מתחיל/ה טיפול לפני קבלת התשובה ההיסטולוגית?

☐ כן

☐ לא

20. מהי בחירתך המועדפת לטיפול קו ראשון ב EoE?

ניתן לסמן יותר מתשובה אחת.

☐ דיאטה

☐ PPI

☐ סטרואידים מקומיים

☐ טיפול ביולוגי

21. במקרה של כישלון טיפול קו ראשון, איזה טיפול היית/ה בוחר/ת כקו שני?

ניתן לסמן יותר מתשובה אחת.

☐ דיאטה

☐ PPI

☐ סטרואידים מקומיים

☐ טיפול ביולוגי

22. איזה טיפול היית/ה ממליץ/ה במקרה של כישלון של שני קווי טיפול?

ניתן לסמן יותר מתשובה אחת.

☐ דיאטה

☐ PPI

☐ סטרואידים מקומיים

☐ טיפול ביולוגי

☐ אף אחד מהנ"ל - הפניה למרפאה ייעודית

23. האם היית/ה מפנה כל מטופל חדש עם EoE להערכת רופא אלרגיה?

☐ כן

☐ לא

24. אם לא, באילו מצבים ספציפיים היית/ה בוחר/ת להפנות מטופל עם EoE לרופא אלרגיה?

____

____

25. אם בחרת/ה בטיפול תזונתי, איזו דיאטת אלימינציה הייתה בחירתך הראשונה?

☐ ONE Food Elimination diet

☐ TWO Food Elimination diet

☐ FOUR Food Elimination diet

☐ SIX Food Elimination diet

26. כיצד היית/ה מגדיר/ה "תגובה לטיפול" ב EoE?

☐ שיפור קליני

☐ שיפור אנדוסקופי

☐ שיפור היסטולוגי

☐ כל הנ"ל

27. כעבור כמה זמן מהתחלת טיפול חדש היית/ה חוזר/ת על גסטרוסקופיה במטופל עם EoE?

☐ חודש

☐ 3 חודשים

☐ חצי שנה

☐ כתלות בקליניקה

28. במטופל EoE אסמפטומטי תחת טיפול, מהי תדירות המעקב האנדוסקופי עליו היית/ה ממליץ/ה?

☐ כל 3 חודשים

☐ כל חצי שנה

☐ כל שנה

☐ אחר

Eosinophilic Esophagitis Survey

EoE

A survey of gastroenterologists on the diagnosis, treatment, and follow-up of patients with eosinophilic esophagitis.

All questions are required in the original form. Select one answer unless multiple selections are indicated.

1. Sex:

☐ Male
☐ Female
☐ Prefer not to answer

2. Which age group do you belong to?

☐ 20-29
☐ 30-39
☐ 40-49
☐ 50-59
☐ Over 60

3. As a gastroenterologist, I treat:

☐ Adults
☐ Children

4. What is your current career stage?

☐ Specialist
☐ Trainee

5. If you are a specialist, how many years have you practiced as a specialist in gastroenterology?

☐ 0-5
☐ 6-10
☐ More than 10

6. Where do you work?

☐ Hospital
☐ Community practice
☐ Both

7. In which region(s) of Israel do you work?

Select all that apply.

☐ North
☐ South
☐ Central Israel
☐ Jerusalem area

8. What are your areas of interest within gastroenterology?

Select all that apply.

☐ Neurogastroenterology
☐ Inflammatory bowel disease
☐ Advanced endoscopy
☐ Nutrition
☐ Hepatology
☐ Malignancy
☐ General gastroenterology

9. On average, how many upper gastrointestinal endoscopies do you perform per month?

☐ Fewer than 10
☐ 10-19
☐ 20-39
☐ 40 or more

10. In which of the following situations would you obtain esophageal biopsies despite a completely normal endoscopic appearance?

Select all that apply.

☐ Dysphagia
☐ Chest pain
☐ Refractory heartburn
☐ Recurrent vomiting
☐ Dyspepsia
☐ Impaired growth
☐ None of the above
☐ Other: ____________________________________

11. Would you obtain esophageal biopsies in a patient with findings suggestive of EoE but no relevant symptoms?

☐ Yes

☐ No

12. Which endoscopic findings would you expect to see in EoE?

Select all that apply.

☐ Normal endoscopic appearance

☐ Esophageal edema

☐ White exudates

☐ Rings

☐ Furrows

☐ Strictures

☐ Ulcers

13. Are you familiar with the EREFS scoring system for the endoscopic assessment of EoE severity?

☐ Yes

☐ No

14. In cases of acute food impaction, do you obtain esophageal biopsies, and if so, when?

☐ During the urgent endoscopy

☐ At a later date

☐ Not at all

15. How many esophageal biopsies do you obtain when EoE is suspected?

☐ 2 biopsies

☐ 4 biopsies

☐ 6 or more biopsies

16. From which sites do you obtain biopsies when EoE is suspected?

Select all that apply.

☐ Distal esophagus

☐ Mid esophagus

☐ Proximal esophagus

☐ Stomach

17. When EoE is suspected and biopsies are obtained from more than one site, how do you submit the specimens?

☐ In a single container

☐ In separate containers

18. Do you recommend discontinuing proton pump inhibitor (PPI) therapy before upper gastrointestinal endoscopy when EoE is suspected?

☐ Yes

☐ No

19. In a patient with an endoscopic appearance suggestive of EoE, would you initiate treatment before receiving the histology results?

☐ Yes

☐ No

20. What is your preferred first-line treatment for EoE?

Select all that apply.

☐ Dietary therapy

☐ PPI

☐ Topical corticosteroids

☐ Biologic therapy

21. If first-line treatment fails, which treatment would you choose as second-line therapy?

Select all that apply.

☐ Dietary therapy

☐ PPI

☐ Topical corticosteroids

☐ Biologic therapy

22. Which treatment would you recommend after failure of two lines of therapy?

Select all that apply.

☐ Dietary therapy

☐ PPI

☐ Topical corticosteroids

☐ Biologic therapy

☐ None of the above - referral to a dedicated clinic

23. Would you refer every newly diagnosed patient with EoE to an allergist for evaluation?

☐ Yes
☐ No

24. If not, in which specific situations would you refer a patient with EoE to an allergist?

____

____

25. If you choose dietary therapy, which elimination diet would be your first choice?

☐ One-food elimination diet
☐ Two-food elimination diet
☐ Four-food elimination diet
☐ Six-food elimination diet

26. How would you define a "response to treatment" in EoE?

☐ Clinical improvement
☐ Endoscopic improvement
☐ Histologic improvement
☐ All of the above

27. How long after initiating a new treatment would you repeat upper gastrointestinal endoscopy in a patient with EoE?

☐ 1 month
☐ 3 months
☐ 6 months
☐ Depending on the clinical presentation

28. For an asymptomatic patient with EoE receiving treatment, how frequently would you recommend endoscopic follow-up?

☐ Every 3 months
☐ Every 6 months
☐ Annually
☐ Other
